# SIRT6 Is a Positive Regulator of Aldose Reductase Expression in U937 and HeLa cells under Osmotic Stress: *In Vitro* and *In Silico* Insights

**DOI:** 10.1371/journal.pone.0161494

**Published:** 2016-08-18

**Authors:** Ahmet Can Timucin, Huveyda Basaga

**Affiliations:** Molecular Biology, Genetics and Bioengineering Program, Faculty of Engineering and Natural Sciences, Sabanci University, Orhanli, Tuzla, Istanbul, Turkey; Institute of Nutrition, GERMANY

## Abstract

SIRT6 is a protein deacetylase, involved in various intracellular processes including suppression of glycolysis and DNA repair. Aldose Reductase (AR), first enzyme of polyol pathway, was proposed to be indirectly associated to these SIRT6 linked processes. Despite these associations, presence of SIRT6 based regulation of AR still remains ambiguous. Thus, regulation of AR expression by SIRT6 was investigated under hyperosmotic stress. A unique model of osmotic stress in U937 cells was used to demonstrate the presence of a potential link between SIRT6 and AR expression. By overexpressing SIRT6 in HeLa cells under hyperosmotic stress, its role on upregulation of AR was revealed. In parallel, increased SIRT6 activity was shown to upregulate AR in U937 cells under hyperosmotic milieu by using pharmacological modulators. Since these modulators also target SIRT1, binding of the inhibitor, Ex-527, specifically to SIRT6 was analyzed *in silico*. Computational observations indicated that Ex-527 may also target SIRT6 active site residues under high salt concentration, thus, validating *in vitro* findings. Based on these evidences, a novel regulatory step by SIRT6, modifying AR expression under hyperosmotic stress was presented and its possible interactions with intracellular machinery was discussed.

## 1. Introduction

A well-established target of the transcription factor NFAT5 (nuclear factor of activated T-cells 5), aldose reductase (AR) is responsible from reduction of various substrates including glucose, as well as, atherogenesis related aldehydes [1–3]. Despite its protective role against hyperosmotic stress, this enzyme was also shown to be involved in several aspects of diabetic vascular complications including neuropathy, nephropathy, retinopathy and atherosclerosis [4–7]. In this regard, research on AR has been focused on inhibition of its enzymatic mechanism, but vast majority of the clinical trials were considered unsuccessful until now [3, 8]. Therefore, an alternative approach towards AR would be the fine tuning of its expression level, using other intracellular effectors [8]. Among these possible factors regulating AR expression, our group recently identified sirtuin1 (SIRT1)—poly-(ADP-ribose) polymerase1 (PARP1) axis regulating NFAT5 dependent AR expression under osmotic stress [9]. Since this axis was shown to be in crosstalk with sirtuin6 (SIRT6) in other experimental settings, it was also proposed that SIRT6 may also regulate AR expression [10, 11].

SIRT6, a nicotinamide adenine dinucleotide (NAD^+^) dependent protein deacetylase, was suggested to regulate wide range of intracellular processes including metabolism and genomic maintenance [12]. In particular, this enzyme was suggested to inhibit glycolysis and was shown to activate PARP1 for DNA repair under stress [10, 11, 13]. Both of these regulatory steps were also previously linked to processes in which AR, the rate limiting enzyme of polyol pathway, also takes part. For instance, inhibition of glycolysis has been suggested to direct glucose to other pathways of glucose utilization, including polyol pathway [14]. Moreover, PARP1 was also suggested to interact and inhibit the transcriptional activity of NFAT5, the transcription factor of AR [15]. Apart from these conceptual links, possible regulation of AR expression by SIRT6 still remains obscure, which prompted this study.

In this study, for the first time, identification of SIRT6 as a regulator of AR expression under hyperosmotic stress, was described. A unique osmotic stress model of U937 cells was primarily used to construct a potential link between SIRT6 and AR. Overexpression of SIRT6 in HeLa cells under hyperosmotic stress were used to display the role of SIRT6 on regulation of AR expression. By employing pharmacological modulators, the role of SIRT6 enzymatic activity on AR expression in osmotic stress model of U937 cells, was also used to confirm *in vitro* findings. Since these modulators were specific for SIRT1, *in silico* molecular simulations were performed to resolve the molecular machinery leading to inhibitor targeted SIRT6 under high salt concentration. Overall, SIRT6 was identified as a novel positive regulator of AR expression under hyperosmotic stress. In view of the evidence provided, possible crosstalk on related signaling pathways was also discussed.

## 2. Materials & Methods

### 2.1. Cell culture, treatments and viability

Cell culture and their treatments were performed as previously described with minor modifications [9]. U937, human histiocytic lymphoma cell line, cultured in RPMI-1640 with 5 mM glucose, 10% FBS, 2 mM glutamine and 100 IU/ml penicillin/streptomycin, in a humidified incubator with an atmosphere of 5% CO_2_, at 37°C [16]. Cells were collected by centrifugation at 300g for 5 minutes, resuspended in serum free medium (SFM) and seeded (1,000,000 cells/ml) in 100 mm culture plates. In experiments with U937 cells, 100 mM NaCl was applied to cells from a 1 M stock prepared in SFM and an equal volume of SFM was added to the control. Osmolality change of the medium was confirmed using an osmometer (Osmomat 030, Gonatech, Berlin, Germany). Pretreatment with Trichostatin A (TSA), NAD^+^ and Ex-527, all of which were dissolved in DMSO, was applied 1 hr prior to applying osmotic stress agent. For each pretreatment experiment, DMSO (max 0.5%, v/v) was added to all controls. HeLa cells were cultured in DMEM supplemented with 5 mM glucose, 10%FBS and 100 IU/ml penicillin/streptomycin, maintained similar to U937 cells [17]. In all HeLa experiments, 100 mM NaCl was applied to cells from 1M stock prepared in culture medium. For cellular viability assay, U937 and HeLa cells were seeded in 96-well plates, treated as indicated and analyzed by MTT Cell Proliferation Kit I according to the manufacturer's instructions.

### 2.2. Antibodies and reagents

RPMI-1640, DMEM, FBS and antibiotics were purchased from Pan Biotech GmbH (Aidenbach, Germany). MTT Cell Proliferation Kit I, X-tremeGENE 9 DNA transfection reagent, protease and phosphatase inhibitor cocktails were purchased from Roche (F. Hoffman-La Roche Ltd., Basel, Switzerland). Primary NFAT5 (sc-13035, rabbit) and AR (sc-166919, mouse) antibodies were purchased from Santa Cruz Biotechnology Inc. (Dallas, Texas, USA) and used with 1:200 dilution in immunoblotting. SIRT6 antibody (SAB4200254, mouse) was purchased from Sigma (Darmstadt, Germany) and used with 1:1000 dilution in immunoblotting. Primary SIRT1 (#8469, mouse), PARP1 (#9542, rabbit), myc (#2272, rabbit), Lamin A/C (#2032, rabbit) and Beta-Actin (#4967, rabbit) antibodies were purchased from Cell Signaling Technology Inc. (Beverly, MA, USA) and used with 1:1000 dilution in immunoblotting. Secondary HRP-conjugated anti-rabbit (#7074) and anti-mouse (#7076) antibodies were also purchased from Cell Signaling Technology Inc. (Beverly, MA, USA) and used with 1:5000 dilution in immunoblotting. NaCl, glucose, tris, glycine, and tween-20 were purchased from Molekula Ltd. (Newcastle Upon Tyne, UK). All other chemicals were obtained from Sigma (Darmstadt, Germany), unless otherwise stated.

### 2.3. Protein extraction and immunoblotting

Protein extraction and immunoblotting protocols were applied as previously described, with minor modifications [9]. For total protein extraction, cells were treated as indicated in 100 mm culture plates and harvested by centrifugation, washed with ice-cold PBS and centrifuged again to obtain cell pellet. Pellet was lysed by total cell lysis buffer, followed by centrifugation. Supernatant was collected as total protein extract and stored in -80°C for immunoblotting analysis. For cytoplasmic-nuclear extraction, cells were treated as indicated in 100 mm culture plates and harvested by centrifugation washed with ice-cold PBS and centrifuged again to obtain cell pellet. For cytoplasmic extraction, pellet was first lysed by incubation in T1 buffer, followed by centrifugation. Supernatant was collected as cytoplasmic extract and stored in -80°C for immunoblotting analysis. For nuclear extraction, remaining pellet was resuspended in T2 buffer, followed by centrifugation. Supernatant was collected as nuclear extract and stored in -80°C for immunoblotting analysis. Protein concentrations were determined by Bio-Rad Protein Assay (Bio-Rad, Munich, Germany) based on Bradford method. Proteins (30–100 μg) were mixed with loading buffer, separated on 6–12% SDS-PAGE and blotted onto PVDF membranes. Membranes were then blocked with 5% non-fat dry milk (AppliChem GmbH, Darmstadt, Germany) in PBS-Tween20, incubated with primary antibody overnight, followed by washing in PBS-Tween20 and incubation with HRP-conjugated secondary antibody. After the final wash with PBS-Tween20, proteins were analyzed with ECL Prime (GE Healthcare Bio-Sciences, UK) and exposed to Lumi-Film Chemiluminescent Detection Film (Roche Diagnostics, Mannheim, Germany). Beta-actin was used as total and cytoplasmic protein loading control, whereas Lamin A/C was used as nuclear loading control. All immunoblotting images were analyzed using ImageJ program [18].

### 2.4. Transfections

Transfections were performed as previously described, with minor modifications [9]. Flag tagged wild-type SIRT6 was a gift from Eric Verdin (Addgene plasmid # 13817) [19]. 6x myc tagged pEGFP NFAT5 (no EGFP) plasmid was a gift from Anjana Rao (Addgene plasmid # 13627) [20]. HeLa cells were transfected either with wild-type flag tagged SIRT6 or with 6x myc tagged pEGFP NFAT5 (no EGFP). All transfection experiments were done using X-tremeGENE 9 DNA transfection reagent, according to the manufacturer`s instructions. Depending on the experiment, empty backbone plasmids were also transfected as negative transfection control and indicated as mock. Transfections were confirmed by immunoblotting of the tag, protein itself or both.

### 2.5. Structures and docking

The crystal structures of SIRT6 (PDB entry: 3K35 Chain A) and of SIRT1 (PDB entry: 4I5I Chain A) were used as the receptor for docking of the ligand, Ex-527. [21, 22]. This crystal structure of SIRT1 was used as a positive control since it contained an analogue of Ex-527 in interaction with hydrophobic pocket amino acids and NAD^+^ [22]. By comparing amino acids of SIRT1 hydrophobic pocket using a previously published alignment of the core deacetylase domains of human SIRTs, the hydrophobic pocket of SIRT6 was predicted and included in the search space during docking [23]. Structure of Ex-527 was obtained from Chemspider database (ID: 4288080) and its parameters were determined using SwissParam [24]. Ex-527 was docked both to the hydrophobic pocket of SIRT1 and SIRT6 using Autodock Vina (version 1.1.2) docking program [25]. Final docking poses were selected based on proximity of Ex-527 to the hydrophobic amino acids and Autodock binding scores.

### 2.6. FoldX calculations

Protein design tool FoldX (version 4) was utilized to assess the effect of hydrophobic residue mutations on the stability of SIRT1 and SIRT6 [26–28]. By comparing wild type to mutant structures, FoldX predicts stability change in proteins based on unfolding free energy difference (ΔΔG). The five hydrophobic residues determined for SIRT6 (PDB entry: 3K35 Chain A), as well as, corresponding residues of SIRT1 (PDB entry: 4I5I Chain A), were individually mutated to alanine or glycine and stability change due to variation in intermolecular interactions, was determined by FoldX. During calculations, the temperature was set to 310K and average of five runs was used to compute the ΔΔG. This method suggests that ΔΔG values >0.5 kcal/mol correspond to destabilizing mutations, while ΔΔG values <-0.5 kcal/mol match to stabilizing mutations. In our case, ΔΔG values obtained from SIRT1 mutations were compared with ΔΔG values of SIRT6 mutations, in part, to validate the presence of a similar hydrophobic pocket in SIRT6.

### 2.7. Molecular dynamics simulations

Molecular dynamics (MD) simulations were performed, as previously described, using the structures obtained from docking step [9]. As the positive control, SIRT1-Ex-527 complex was used in MD simulations. The SIRT1-Ex-527 and SIRT6-Ex-527 complex, composed of 4346 and 4342 atoms were placed in water boxes with dimensions of 75x53x55 and 85x67x60 Å^3^, respectively. Then the systems were neutralized with NaCl at 10 mM or 100 mM of final concentrations. Simulations completed in 10 mM were referred as low salt (LS) while simulations in 100 mM NaCl, were referred as high salt (HS). Simulations under LS were utilized to control for the outcomes of the simulations done under HS environment. The resulting systems were used in MD simulations using the NAMD program [29] with the CHARMM22 parameters [30, 31] which included correction map (CMAP) for backbone atoms [32, 33]. Water molecules within the system were treated explicitly using the TIP3P model [34]. An NpT ensemble was used in MD simulations with periodic boundary conditions, and the long-range Coulomb interactions were computed using the particle-mesh Ewald algorithm. Pressure was maintained at 1 atm and temperature was maintained at 310 K using the Langevin pressure and temperature coupling. A time step of 2 fs was used in all MD simulations. The systems were fully energy minimized in 20,000 steps, followed by heating slowly from 10 K to 310 K in 30 ps. Then they were carefully equilibrated under constant temperature and volume for 0.5 ns before production runs. The production was lasted for 20 ns and repeated twice. Visual molecular dynamics (VMD) [35] was used for the analysis of trajectories and the visualization of structures. Root mean square displacements (RMSD) for the backbone atoms (C, N, Cα) of each protein were analyzed for stability. Residue-wise root mean square fluctuations (RMSF) of Ex-527 heavy atoms, excluding hydrogens, were measured for flexibility analysis of the ligand. Distance between Ex-527 and hydrophobic pockets were computed to differentiate if the ligand was held in close proximity to the pockets during 20 ns. LigPlot^+^ (version 1.4) tool was used to evaluate the interactions under high salt condition at the 20th ns of MD simulations. [36]. Distances from Ligplot^+^ selected Ex-527 atoms to the Ligplot^+^ selected atoms of molecules interacting with Ex-527 at 20^th^ ns of MD simulations, were measured throughout the simulations completed under high salt environment, to present the variation of Ex-527 dynamics.

### 2.8. Statistical analyses

All *in vitro* results were representative of at least three independent experiments, while *in silico* results were representative of two. All numerical data were expressed as mean ± SEM. Statistical significance was determined using student’s t-test. p value of < 0.05 was accepted as statistically significant.

## 3. Results

### 3.1. SIRT6 positively regulates AR expression under hyperosmotic stress

**Fig 1:**
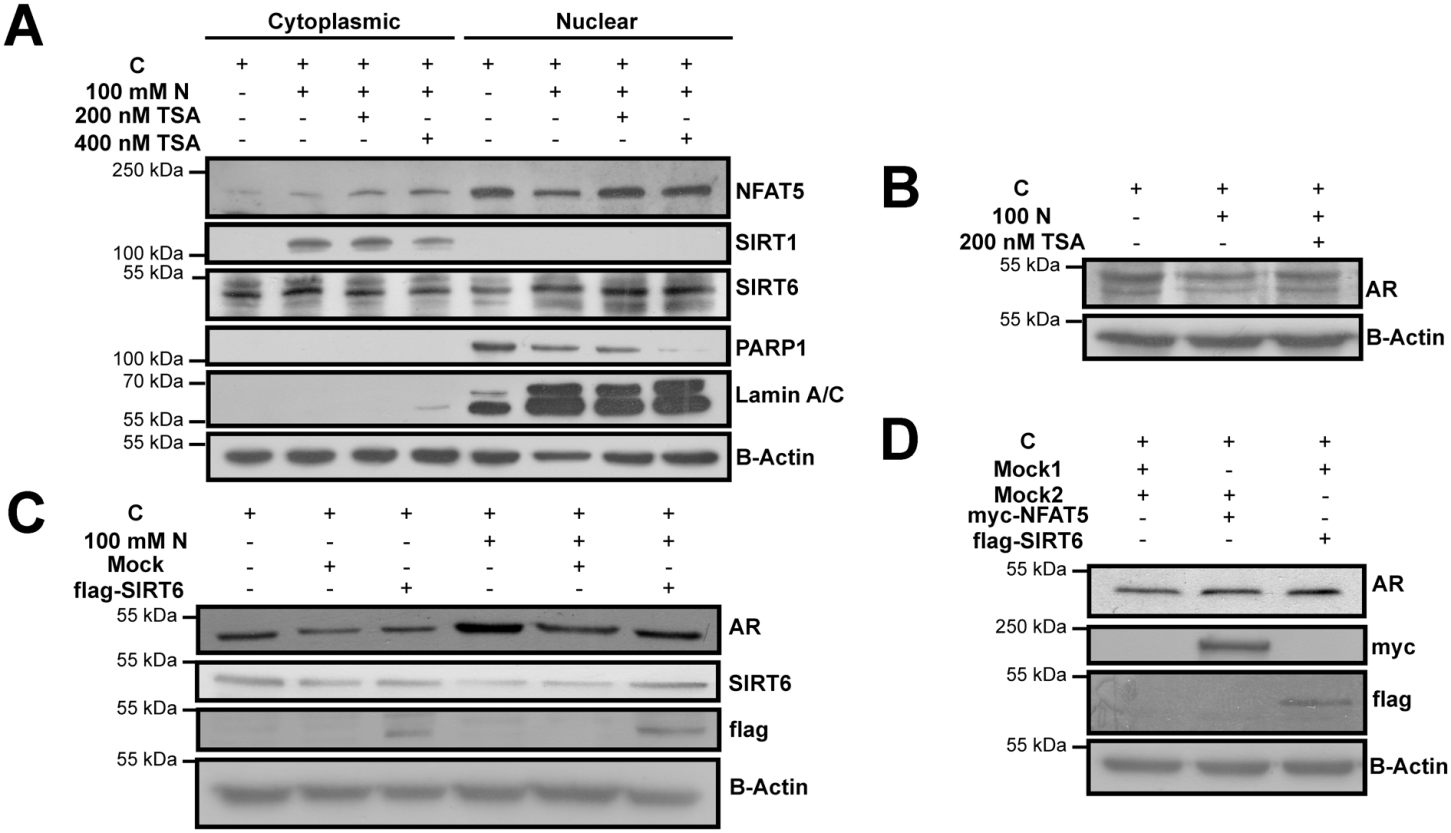
SIRT6 upregulated AR expression under hyperosmotic stress. Control (C) indicates U937 cells treated with 5 mM glucose containing SFM for 16 hrs in (A) and (B). 100 mM N indicates U937 cells further treated with 100 mM NaCl (N) for 16 hrs in (A) and (B). U937 cells were pretreated either with solvent (DMSO) or indicated concentrations of TSA for 1 hr, followed by 16 hrs of 100 mM N treatment in (A) and (B). (A) (B) 200 nM TSA pretreatment increased nuclear NFAT5, nuclear SIRT6 and total AR expressions, without altering SIRT1 and PARP1 expressions in U937 cells under hyperosmotic stress. Control (C) indicates HeLa cells treated serum containing medium with 5 mM glucose in (C) and (D). 100 mM N indicates HeLa cells further treated with 100 mM NaCl (N) for 16 hrs in (C). (C) Overexpression of wildtype flag tagged SIRT6 enhanced AR expression both under normosmotic and hyperosmotic conditions in HeLa cells, compared with mock controls. (D) Overexpression of wildtype flag tagged SIRT6 and overexpression of myc-NFAT5 alone displayed similarly increased AR expression, compared with the mock control in HeLa cells. Expressions were evaluated using immunoblotting in cytoplasmic and nuclear extracts in (A) and in total extracts in (B), (C) & (D). Expression of SIRT6, flag and myc was used for confirmation of overexpression. Mock1 indicates the backbone plasmid of myc-NFAT5 and mock2 indicates backbone plasmid of flag-SIRT6. Results obtained from this figure was based on the densitometry based statistical analyses, given in S2 and S3 Figs.

A unique model of hyperosmotic stress, recently characterized by our laboratory for its non-canonical osmotic stress response, was used to construct the main frame of this study [9]. This model, 16 hrs of 100 mM NaCl treatment to U937 cells, resulted in higher SIRT6 but lower NFAT5 and PARP1 expressions in nucleus, increased level of SIRT1 expression in cytoplasm and diminished total AR expression, compared with normosmotic control (p < 0.05) (Fig 1A, Lanes 1, 2, 5 and 6 and S2 Fig & Fig 1B Lanes 1 and 2 and S3A Fig) [9]. In order to control for excessive cell death that might interfere with the data obtained, 100 mM NaCl treatment was also analyzed in terms of cellular viability in U937 and HeLa cells (S1 Fig). 16 hrs of 100 mM NaCl treatment diminished metabolic activity in a statistically insignificant manner, in both cell lines, indicating 100 mM NaCl treatment may be used for further analysis (p > 0.05) (S1 Fig).

In order to decipher the variation in the expression of SIRT6 when NFAT5 dependent AR expression was upregulated in this model, pretreatment of TSA, an agent that has been shown to induce binding of NFAT5 to its target promoter sites and to upregulate its target genes, was utilized [37, 38]. Remarkably, at 200 nM TSA pretreatment, an increased nuclear SIRT6 expression was accompanied by increased nuclear NFAT5 and total AR expressions, compared with hyperosmotic stress alone (p < 0.05) (Fig 1A Lanes 6 and 7 and S2A and S2C Fig and Fig 1B Lanes 2 and 3 and S3A Fig). Since NFAT5 dependent AR expression was recently suggested to be under SIRT1-PARP1 interplay, expressions of SIRT1 and PARP1 were also analyzed after TSA pretreatments [9]. Convincingly, SIRT1 and PARP1 expressions remained constant after 200 nM TSA treatment but not after 400 nM TSA (p < 0.05), indicating the likelihood of crosstalk from SIRT1-PARP1 axis was minimal at the former under osmotic stress (Fig 1A, Lanes 2, 3, 4, 6, 7 and 8 and S2B and S2D Fig). Based on these results, it was hypothesized that SIRT6 could be considered as a factor influencing AR expression under hyperosmotic stress.

With the purpose of testing this hypothesis, wildtype flag tagged SIRT6 was overexpressed in HeLa cells, followed by similar hyperosmotic stress treatment, and AR expression was explored (Figs 1C and S3B). It was evident that overexpression of SIRT6 yielded more AR expression independent of the stress, compared with mock control (p < 0.05) (Fig 1C Lanes 2, 3, 5, 6 and S3B Fig). In order to validate this observation, overexpression of myc-NFAT5, a mimicry of hyperosmotic stress, was compared with overexpression of flag-SIRT6 alone in terms of their contribution to AR expression in HeLa cells (Figs 1D and S3C). Similarly, both overexpression of NFAT5 and SIRT6 displayed equivalently increased AR expression compared with mock control (p < 0.05) (Figs 1D and S3C). Hence, it was concluded that SIRT6 may be considered as an inducer of AR expression, regardless of the stress condition. Since our hypothesis was constructed upon a hyperosmotic condition, detailed emphasis were given to the data obtained under osmotic stress in the rest of the manuscript.

### 3.2. Increased SIRT6 activity upregulates nuclear SIRT6 and total AR expression under hyperosmotic stress

**Fig 2:**
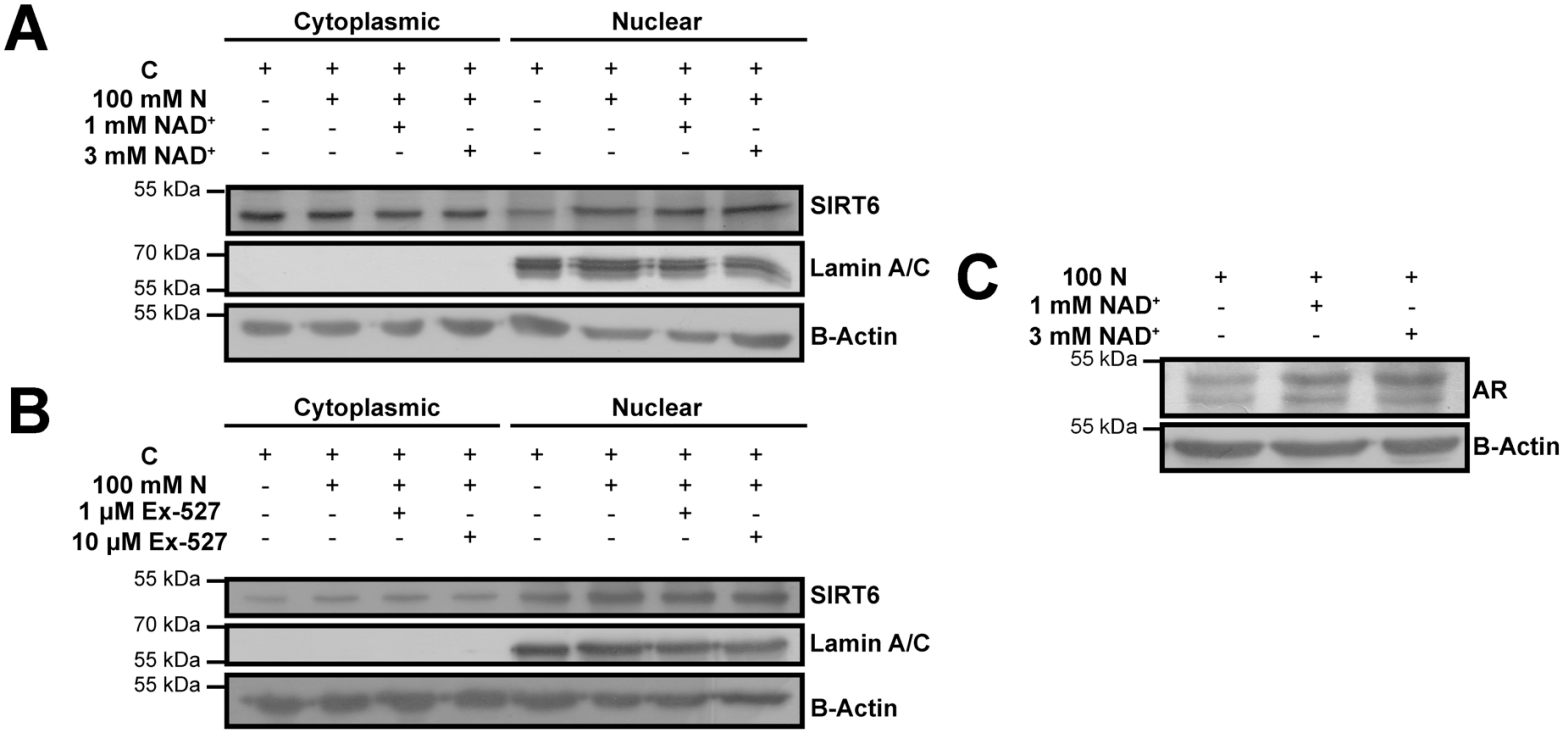
The cofactor of SIRT6, NAD^+^ positively regulated nuclear SIRT6 accumulation and total AR expression under hyperosmotic stress. Control (C) indicates U937 cells treated with 5 mM glucose containing SFM for 16 hrs in (A) and (B). 100 mM N indicates U937 cells further treated with 100 mM NaCl (N) for 16 hrs in (A), (B) and (C). U937 cells were pretreated either with solvent (DMSO) or indicated concentrations of NAD^+^ or Ex-527 for 1 hr, followed by 16 hrs of 100 mM N treatment in (A), (B) and (C). (A)(C) The cofactor of SIRT6, NAD^+^, enhanced the nuclear accumulation of SIRT6 and total AR expression in dose-dependent manner under hyperosmotic stress. (B) A specific SIRT1 inhibitor and recently suggested SIRT6 inhibitor, Ex-527 did not altered nuclear SIRT6 expression under hyperosmotic stress, in part, justifying the data obtained in (A). Expressions were evaluated using immunoblotting in cytoplasmic and nuclear extracts in (A) & (B) and in total extracts in (C). Results obtained from this figure was based on the densitometry based statistical analyses, given in S4 Fig.

For further validation of the role of SIRT6 as an activator of AR expression, the mechanism of this regulation was investigated in terms of SIRT6 activity. In order to delineate this role, activator/inhibitor pretreatment studies prior to hyperosmotic stress were conducted. Since, there were no previously identified or commercially available specific SIRT6 pharmacological modulators, previously utilized cofactor of SIRT6, NAD^+^, was used as activator [21]. Treatment with NAD^+^ prior to hyperosmotic stress demonstrated that more SIRT6 accumulated to nucleus and total AR expression was upregulated, compared with hyperosmotic stress only control (p < 0.05) (Fig 2A Lanes 6, 7 and 8 and S4A Fig & Fig 2C Lanes 1, 2 and 3 and S4C Fig). Accordingly, it was reasoned that increased enzymatic activity of SIRT6 could be involved in increased nuclear localization of SIRT6 and upregulation of AR expression.

To confirm the outcome obtained from NAD^+^ pretreatment experiments, a well-established SIRT1 inhibitor which was also recently identified as SIRT6 inhibitor, Ex-527, was employed [39]. Pretreatment with Ex-527 displayed no change in nuclear accumulation of SIRT6 under osmotic stress, compared with hyperosmotic stress only control (Fig 2B Lanes 6, 7 and 8 and S4B Fig). This observation was also concordant with the finding on our recently published data, signifying that Ex-527 prevents AR expression under hyperosmotic stress [9]. Thus, data from Ex-527 pretreatment was considered to justify the observations gained from NAD^+^ pretreatment under hyperosmotic stress.

Moreover, increased SIRT6 expression in osmotic stress only group compared with normosmotic control in S4A and S4B Fig clearly validated the observation of increased SIRT6 expression of hyperosmotic stress model shown in Figs 1A and S2C.

Together, it was evident that enzymatic activity of SIRT6 positively regulated its own nuclear accumulation in parallel to upregulation of AR expression. Nevertheless, the pharmacological modulators utilized in these studies did not only target SIRT6 but also targeted SIRT1 [40]. Thus, *in silico* studies were conducted to demonstrate specific targeting of SIRT6 by the inhibitor, Ex-527, under hyperosmotic milieu.

### 3.3. *In silico* prediction of SIRT6 as a plausible Ex-527 target

**Fig 3:**
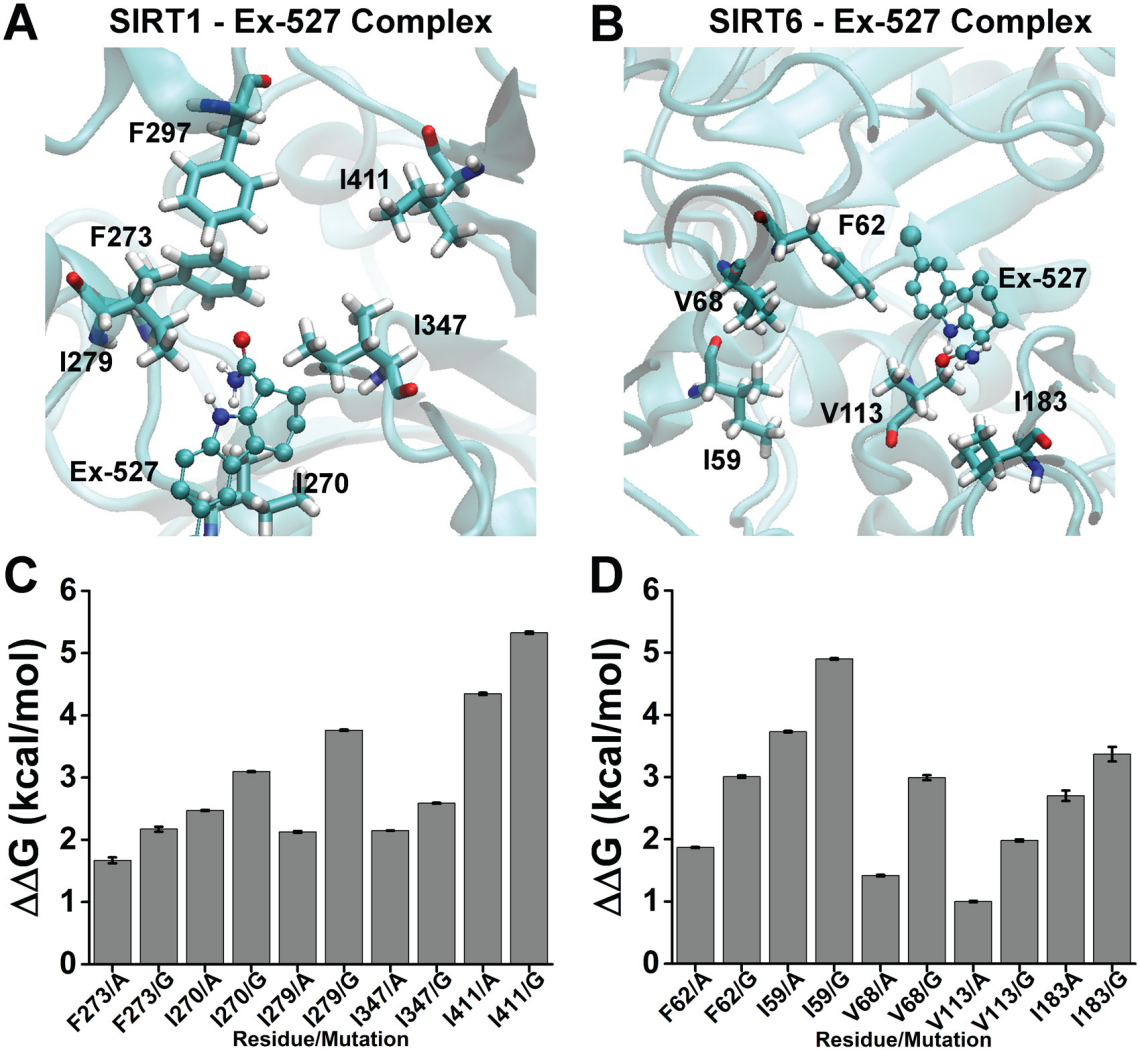
Hydrophobic pockets of SIRT1 and SIRT6. Complexes obtained by docking the inhibitor, Ex-527, to the close proximity of hydrophobic pocket of SIRT1 (A) and to the analogous hydrophobic pocket of SIRT6 (B), were used to simulate the permenance of the binding of the inhibitor to the hydrophobic cluster amino acids. Each amino acid was shown for clear positioning of the pockets. SIRT6 hydrophobic pocket amino acids (B) were predicted based on the corresponding amino acids of SIRT1 (A): V113, I183, I59, F62, V68 of SIRT6 were selected based on similarity to I347, I411, I270, F273, I279 of SIRT1. F297, a member of SIRT1 hydrophobic pocket shown in (A), does not have a corresponding hydrophobic residue in SIRT6 structure (B). (C)(D) Alanine and glycine mutations of SIRT1 hydrophobic pocket residues showed destabilizing characteristic (positive ΔΔG values) similar to the mutations of SIRT6, in part, validating the presence of a hydrophobic pocket in SIRT6 structure. FoldX was used to compute ΔΔG values to determine stability change. Protein structures obtained from PDB ID: 4I5I chain A and PDB ID: 3K35 chain A were used as SIRT1 and SIRT6, respectively.

Since targeting of Ex-527 on SIRT6 was suggested once through an *in vitro* model with lysine 56 of histone H3 as the substrate, it was questioned if Ex-527 directly targeted SIRT6 *in silico* under hyperosmotic environment [39]. In order to understand this targeting mechanism, Ex-527 was docked to the hydrophobic pocket amino acids of SIRT6 and also to those of SIRT1, as previously described (Fig 3A and 3B) [22]. Since Ex-527 was more extensively characterized inhibitor for SIRT1 than SIRT6, SIRT1-Ex-527 complex was used as the positive control in docking studies. It was apparent that similar hydrophobic pockets that were made up of similar hydrophobic amino acids both in SIRT1 and SIRT6, could hold Ex-527 in close proximity after docking (Fig 3A and 3B). In order to validate presence of a hydrophobic pocket in SIRT6 comparable to that of SIRT1, amino acids both in SIRT1 and SIRT6 pockets were mutated into smaller amino acids, (glycine and alanine) and stability change was calculated using FoldX (Fig 3C and 3D). It was clear that mutating hydrophobic pocket residues induced destabilization both in SIRT1 and SIRT6 structure, in part confirming the presence of a similar pocket in SIRT6 (Fig 3C and 3D).

**Fig 4:**
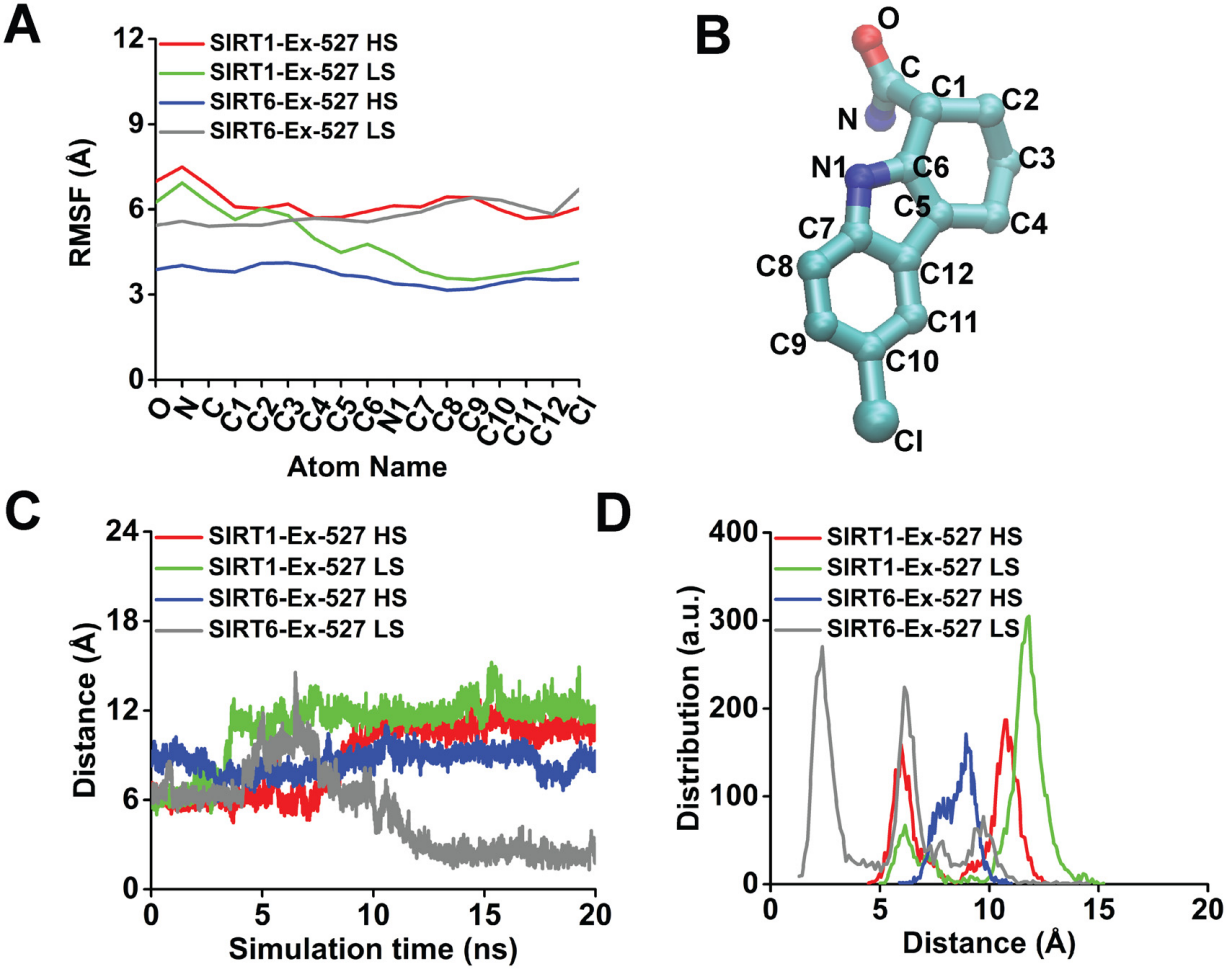
Ex-527 docked to SIRT6, displayed less flexibility and remained in closer proximity to the hydrophobic pocket compared with Ex-527 of SIRT1. (A) RMSF of Ex-527 atoms in SIRT6-Ex-527 complex under high salt (HS) conditions (blue) showed lower flexibility, compared with its positive control, Ex-527 of SIRT1-Ex-527 complex (red) during 20 ns of MD simulations. Under low salt, Ex-527 of SIRT1 (green) had shown lower flexibility than Ex-527 of SIRT6 (gray), for most of the residues. (B) Name of all atoms of Ex-527 were given for clear representation of the results in (A). (C) The distance between the center of mass of Ex-527 and the center of mass of the hydrophobic pocket residues of SIRT6 under high salt concentration (blue) persisted around 7 to 10 Å range, while its corresponding positive control (red) had a clear shift from 6 to 12 Å after ~7 ns of MD simulations. Under low salt, distance between Ex-527 to SIRT6 (gray) even got shortened, while that of SIRT1 (green) was lengthened, compared with Ex-527 of SIRT6 under high salt (blue). (D) Distance distribution vs. distance of Ex-527 to the hydrophobic pockets of all complexes in all simulations were given for clear representation of the distance shifts during the MD simulations in (C). a.u. refers to the arbitrary units, indicating the number of times that each distance was encountered during the simulations. Results obtained from this figure was based on the trends shown and statistical analyses given in S6 Fig. Å: Angstrom, C: Carbon, O: Oxygen, N: Nitrogen, Cl: Chloride

To compare the adjacency of Ex-527 to the hydrophobic pocket residues of SIRT1 and SIRT6, both SIRT1-Ex-527 and SIRT6-Ex-527 complexes were simulated under low salt and high salt conditions for 20 ns using MD. It was clear that RMSD of protein backbone atoms of all of the four simulations reached to a steady plateau, indicating stable simulations for analysis (S5 Fig). In order to explore if Ex-527 was held in close proximity to the hydrophobic pockets, fluctuations of the Ex-527 atoms, the distance between the center of mass of Ex-527 and the center of mass of hydrophobic pockets, and the distribution of these distances were analyzed for all simulations (Figs 4A, 4C, 4D and S6). RMSF analysis indicated that Ex-527 bound to SIRT6 was the least flexible in high salt environment according to RMSF trends of all atoms of Ex-527 (Fig 4A (blue) and 4B) and to the RMSF of C atom of Ex-527 (p < 0.05) (S6A Fig). The distance analysis showed that Ex-527 of SIRT6 under high salt resided mostly within 7 to 10 Å range of SIRT6 hydrophobic pocket during 20 ns of MD simulations (Fig 4C and 4D, blue). Positive control, Ex-527 of SIRT1 under high salt had shown significantly higher fluctuation according to C atom (p < 0.05) (S6A Fig) and larger distance shift (6 to 12 Å) throughout the simulation (Fig 4A, 4C and 4D, blue vs. red) and specifically at 20^th^ ns (p <0.05) (S6B Fig) compared with SIRT6-Ex-527 complex under high salt. Thus, SIRT6 might be a better target of Ex-527 than SIRT1 under hyperosmotic environment. Under low salt, Ex-527 also preferred SIRT6 pocket compared with that of SIRT1. Ex-527 of SIRT1 under low salt conditions had shown comparable flexibility to the Ex-527 of SIRT6 under high salt according to the RMSF of Cl atom of Ex-527 (p < 0.05) (S6C Fig) (Fig 4A, green vs blue). However, this flexibility was mostly achieved through the residues away from hydrophobic pocket since distance of Ex-527 to the hydrophobic pocket had shifted from 6 to 14 Å after 4^th^ ns of the simulations (Fig 4C and 4D, green). This distance was also significantly higher compared with the distance obtained from simulations of SIRT6-Ex-527 complex under high salt at 20^th^ ns (p < 0.05) (S6B Fig). Instead, Ex-527 of SIRT6 under low salt displayed stable flexibility (Fig 4A), got into closer proximity of the hydrophobic pocket after 10^th^ ns (Fig 4C and 4D, gray). Moreover, Ex-527 of SIRT6 under low salt displayed significantly lower distance to hydrophobic pocket compared with Ex-527 of SIRT6 under high salt at 20^th^ ns (p < 0.05) (S6B Fig). Hence, it was apparent that Ex-527 may also target SIRT6 independent of stress (Fig 4A, 4C and 4D, gray).

**Fig 5:**
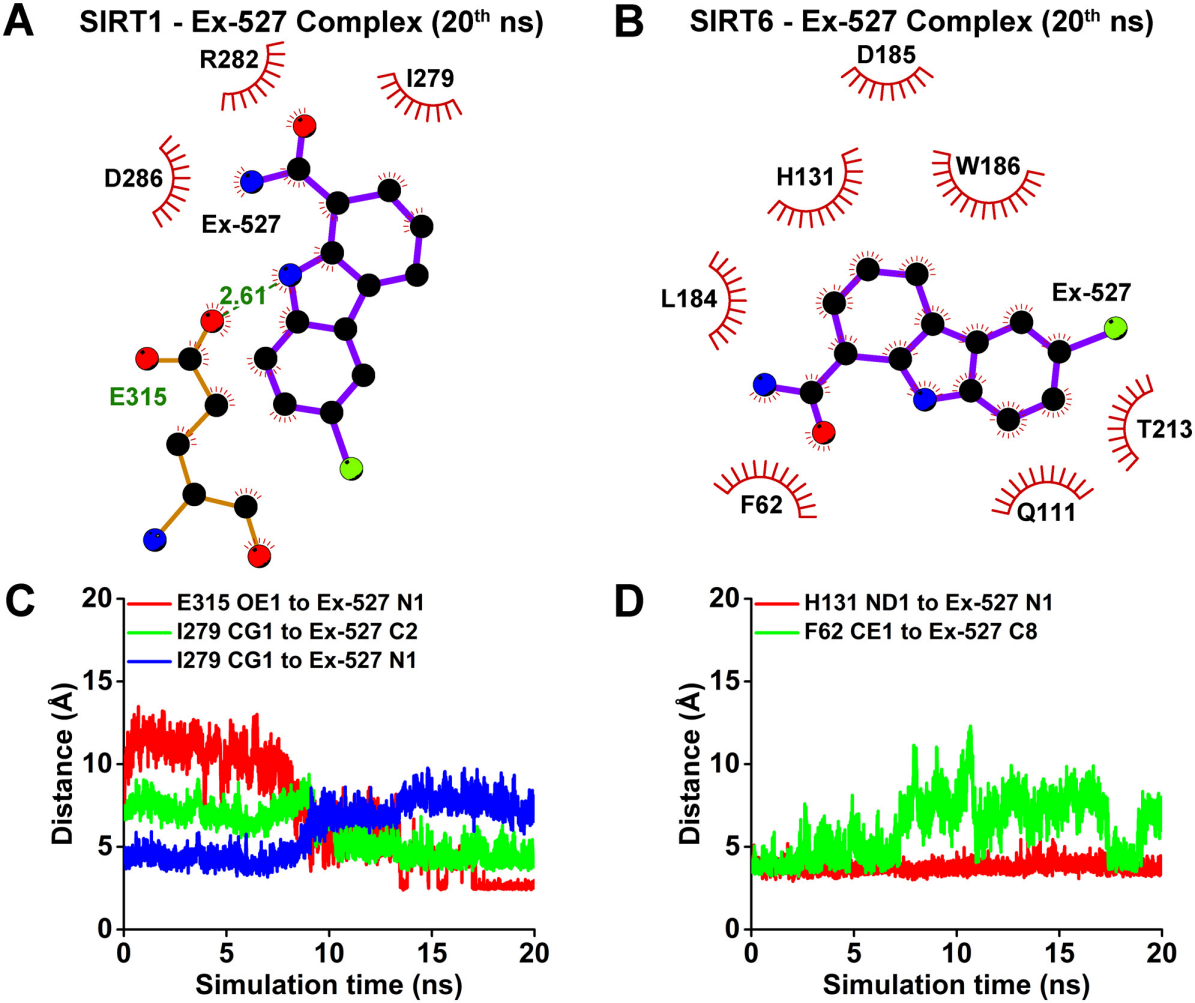
Ex-527 had more hydrophobic contacts and interacted mainly with SIRT6 active site residues compared with Ex-527 of SIRT1. Interaction schemes in (A) and (B) were obtained using Ligplot^+^ interaction analysis tool on the complex structures of SIRT1-Ex-527 and SIRT6-Ex-527 under high salt at the 20^th^ ns of MD simulations. Legend for the items in (A), (B), (C) and (D) were given as supplementary information (S7 Fig). (A) Ex-527 had only one hydrophobic contact remained with the previously established hydrophobic pocket (I279) and it was held close to the hydrophobic pocket with two other hydrophobic contacts (R282, D286) together with a hydrogen bond to E315 (Bond Length: 2.61 Å). (B) Ex-527 of SIRT6 had more hydrophobic contacts than that of SIRT1 and these contacts were made almost completely with SIRT6 active site residues (H131, W186, F62, Q111, T213). Distance vs. time analysis of the selected contacts observed in (A) and (B) for the whole simulations were given as in (C) and (D), respectively. (C) Side chain carbon (CG1) of I279 of SIRT1 was closer to N1 of Ex-527 for first ~7 ns (blue), while it was nearer to C2 of Ex-527 for the rest of the simulation (green). After ~14 ns, side chain oxygen (OE1) of E315 of SIRT1 was in close proximity of N1 of Ex-527, corresponding to the newly formed hydrogen bond (red). (D) For the first ~6 ns, side chain carbon (CE1) of F62 of SIRT6 was closer to the C8 of Ex-527, but this adjacency was fluctuated for the rest of the simulation (green). Yet, active site H131 of SIRT6 was in close proximity to Ex-527 throughout the simulation, presented with distance of its side chain nitrogen (ND1) to N1 of Ex527 (red). Naming for Ex-527 atoms were given in Fig 4B.

In order to comprehend the atomistic details of MD simulations under high salt environment, interaction analyses at 20^th^ ns were utilized and selected distances from the interaction schemes were investigated throughout the simulations. Parallel to the findings in Fig 4C and 4D, Ex-527 of SIRT1 was away from most of the hydrophobic pocket residues except I279 at 20^th^ ns of MD simulations (Fig 5A). Still, it was held as close as possible to the hydrophobic pocket via three hydrophobic contacts (I279, R282, D286) and a single hydrogen bond at 20^th^ ns (Fig 5A). Distance analysis from the selected side chain atom CG1 of I279 to N1 and C2 of Ex-527, indicated that different atoms of Ex-527 may be responsible for hydrophobic contacts to I279 throughout simulations (Fig 5C). Besides, Ex-527 was held as close as possible to the hydrophobic pocket with a hydrogen bond from OE1 of E315 to N1 of Ex-527, formed after ~14^th^ ns (Fig 5A and 5C). Contrary to the observations of SIRT1, Ex-527 of SIRT6 displayed more hydrophobic contacts compared with Ex-527 of SIRT1 and these contacts were made almost completely with active site residues of SIRT6 (H131, W186, F62, Q111, T213) at 20^th^ ns of MD simulations (Fig 5B) [21]. Distance analysis from the selected side chain atom, CE1 of F62 to C8 of Ex-527 showed that these atoms were closer for the first ~6 ns, but their distance was fluctuated in the rest of the simulation (Fig 5D). Albeit the divergence of Ex-527 from hydrophobic pocket member F62, the distance from ND1 of H131 to N1 of Ex-527 was stable throughout 20 ns of MD simulations (Fig 5D).

Overall, it was concluded that Ex-527 may target SIRT6 *in silico*, justifying the results obtained through overexpression and *in vitro* inhibitor studies (Figs 1C, 1D and 2B).

## 4. Discussion

Utilization of a unique hyperosmotic stress model was the initial step to construct the main frame of this study [9]. The underlying motivation for this approach was two folds. First, SIRT1 activity has been suggested to reciprocally regulate its own expression in this model [9, 41]. Therefore, SIRT1 activity, a possible interference to NAD^+^ dependent SIRT6 activity, may be in part controlled by analysis of SIRT1 expression. Second, PARP1, also a NAD^+^ utilizer and a repressor of NFAT5, was suggested to coordinate NFAT5 dependent AR expression in crosstalk with SIRT1 in this model [9, 15, 42]. Based on these previous observations, it was deduced that any change in SIRT6 expression parallel to a change in AR expression, when SIRT1 and PARP1 expressions were held constant, may be considered as a potential link from SIRT6 to AR. This model together with 200 nM TSA pretreatment opened up such a window, and led us to hypothesize the possible role of SIRT6 on AR expression (Fig 1A and 1B).

Overexpression of SIRT6 in HeLa cells clearly identified the role of SIRT6 on AR expression (Fig 1C and 1D). Based on previous literature, this observation may have four novel implications for understanding the SIRT6 based molecular regulation of hyperosmotic stress response. I) Under stress, SIRT6 has been suggested to activate the DNA double strand break repair enzyme, PARP1, a repressor of NFAT5 [11, 15]. Since DNA damage has been suggested to occur in gene deserts as double strand breaks under hyperosmotic stress, SIRT6 might divert PARP1 to these gene free DNA damage sites and allow for more PARP1-free NFAT5 for transcription [43]. II) Another explanation would be the suppression of glycolysis by SIRT6 [10, 13]. Under suppressed glycolysis, excess glucose was shown to be directed to the polyol pathway, which AR constitutes the rate limiting step [14]. Therefore, as a response, cells may have expressed more AR for survival. III) SIRT1 was known to bind to the SIRT6 promoter, which was shown to result in suppression of glycolysis [10]. Therefore, SIRT6 based induction of AR expression through glycolytic suppression may also be mediated by SIRT1. IV) Finally, it is important to note that SIRT6 may directly induce several post translational modifications including deacetylation and/or depalmitoylation on NFAT5 for upregulation of AR [12]. One intriguing example would be the case of depalmitoylation of NFAT5, which resulted in its nuclear translocation [44]. Since hydrolysis of long chain fatty acids by SIRT6 was suggested to increase its deacetylase activity and deacetylation has been suggested to activate NFAT5, SIRT6 may also be considered as direct factor regulating NFAT5 and AR [9, 12]. Overall, each of these explanations or their combinations could orchestrate as the regulatory steps of SIRT6 based regulation of AR, and should be verified experimentally.

Investigation of the contribution of SIRT6 activity to AR expression was one the bottlenecks of this study since there were no commercially available, specific activator or inhibitor for SIRT6. To circumvent this case, previously identified modulators, NAD^+^ and Ex-527 were employed in pretreatment experiments, which displayed results towards the involvement of SIRT6 activity on AR expression (Fig 2A and 2C) [21, 39]. Since these agents also target SIRT1, *in silico* experiments were designed to rationalize the results obtained with *in vitro* inhibitor study (Figs 3–5). Interestingly, SIRT6 displayed even better performance *in silico* than the specific target of Ex-527, SIRT1 (Figs 4 and 5). This might be well explained by the fact that Ex-527 is required to interact with NAD^+^ in order to be held in SIRT1 hydrophobic pocket [22, 45]. Since, the interaction of NAD^+^ with SIRT6 was previously claimed to be considerably divergent compared with other sirtuins, here it was speculated that Ex-527 binding to SIRT6 might not require NAD^+^ as exhibited through *in silico* studies [21]. Furthermore, detailed *in silico* molecular analysis of the interaction scheme and distances selected from these schemes may also hold novel insights towards understanding differential targeting of SIRT1 and SIRT6 by Ex-527 (Fig 5). The change in I279 of SIRT1 contacting residues of Ex-527 may represent the divergence of the ligand from the hydrophobic pocket residues since it was followed with formation of new hydrogen bond between E315 and Ex-527 (Fig 5A and 5C). These series of events may also resemble a case of an alternative binding, in which Ex-527 was held as close as possible to its target hydrophobic pocket with a single hydrogen bond and fewer number of hydrophobic contacts until NAD^+^ is available (Fig 5A and 5C). For the case of SIRT6, F62, a member of hydrophobic pocket, was found to form closer contact with the ligand for the first few ns of the simulations (Fig 5B and 5D). Thus, it can be deduced that F62, as well as other members of SIRT6 hydrophobic pocket may be responsible from handling the inhibitor for initial recognition (Fig 5B and 5D). After recognition was accomplished, H131 of SIRT6, as well as, other active site residues, may be in charge of holding it intact in SIRT6 active site, indicating a NAD^+^ free binding mode of Ex-527 (Fig 5B and 5D). Despite these possible explanations, *in silico* improvement of the discrepancy of SIRT1 being secondary to SIRT6 as a target of Ex-527 should be extensively investigated by utilizing NAD^+^ within the docking, MD, as well as, *in vitro* studies in future. Until then, since *in silico* results were confirmatory of *in vitro*, computational data were considered as informative.

AR and its transcription factor NFAT5 have been linked to several different disease states including cancer and atherosclerosis [46, 47]. It is noteworthy to emphasize here that a potential regulation of NFAT5 and AR by SIRT6 may have important implications on such diseases. For instance, given that the cell lines used U937 and HeLa cells are *in vitro* models of cancer [16, 17, 46], possible regulation of NFAT5 by SIRT6, may translate into discovery of other novel regulatory steps leading to cancer. Moreover, since U937 monocytes were widely used as a model of ox-LDL loaded foam cells of atherosclerotic lesions [47–49], modulation of AR expression by SIRT6 could be used as an experimental method for modulation of atherogenesis, as well. Hence, the findings exhibited through the cell lines utilized, may in turn have possible medical outcomes in future.

**Fig 6:**
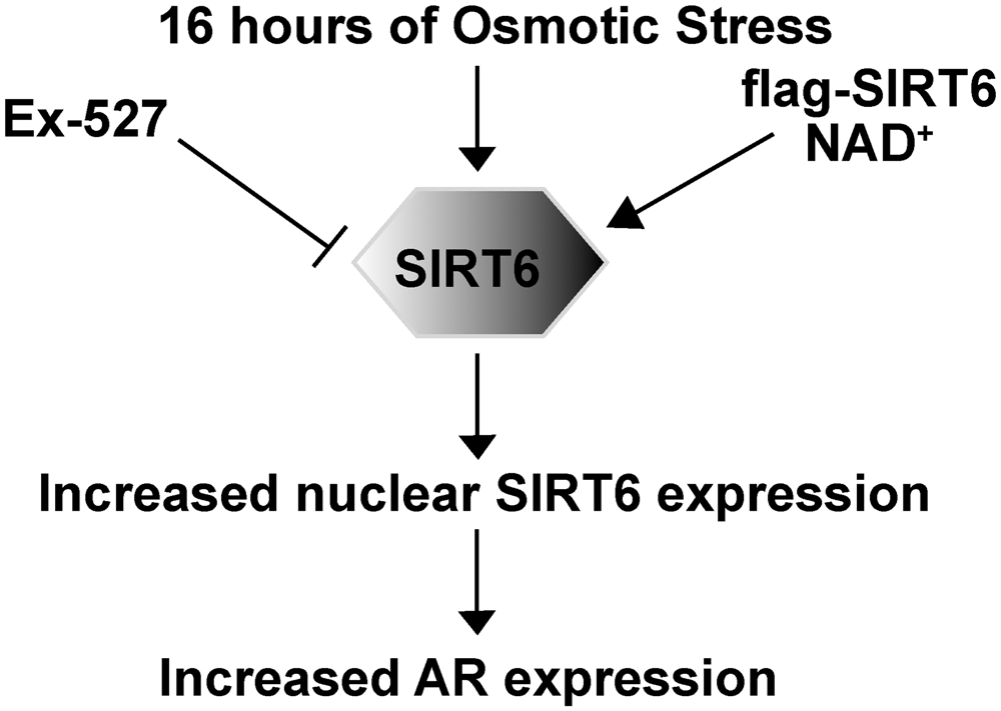
Proposed mechanism for SIRT6 based regulation of AR expression under hyperosmotic environment.

Taken together, here a distinct mechanism of SIRT6, positively regulating AR expression, was proposed (Fig 6). Increased SIRT6 expression through wildtype flag tagged SIRT6 overexpression in HeLa cells yielded increased AR expression under 16 hrs of hyperosmotic stress. This regulation was shown to be linked to increased SIRT6 activity by utilization of pharmacological modulators, NAD^+^ and Ex-527, in U937 cells under hyperosmotic milieu. Since these modulators were also specific for SIRT1, binding of Ex-527 to SIRT6 active site was exhibited using an *in silico* approach.

## 5. Conclusion

For the first time in literature, SIRT6 was identified as an activator of AR expression under hyperosmotic stress. The role of SIRT6 activity on upregulation of AR expression was revealed through pharmacological modulators. Among these modulators, the inhibitor, Ex-527, was analyzed *in silico* and was shown to target SIRT6 active site.

## Supporting Information

S1 Fig 16 hrs of 100 mM NaCl treatment reduced metabolic activity, a marker of cell viability, compared with normosmotic control, in U937 and HeLa cells. Control (C) indicates U937 cells treated with 5 mM glucose containing SFM for 16 hrs and HeLa cells treated with serum containing medium with 5 mM glucose for 16 hrs. 100 mM N indicates U937 and HeLa cells further treated with 100 mM NaCl (N) for 16 hrs. The reduction in cell viability (~13% for U937 and ~15% for HeLa) was not statistically significant in both cell lines (p > 0.05). Metabolic activity status was analyzed by MTT based colorimetric assay. Average absorbance values of the controls were set to 100%.
(TIF)

S2 Fig Densitometry analyses for Fig 1A. Nuclear NFAT5, SIRT6, PARP1 and cytoplasmic SIRT1 expressions were analyzed using densitometry for Fig 1A. Beta-actin (B-actin) was used as cytoplasmic loading control, whereas Lamin A/C (Lamin) was used as nuclear loading control. * indicates statistically significant difference vs C group (p < 0.05). # indicates statistically significant difference vs 100 N group (p < 0.05). N: NaCl.
(TIF)

S3 Fig Densitometry analyses for Fig 1B, 1C and 1D. Total AR expressions were analyzed using densitometry for Fig 1B, 1C and 1D. (A), (B) and (C) corresponds to Fig 1B, 1C and 1D, respectively. Beta-actin (B-actin) was used as total protein loading control. * indicates statistically significant difference vs C group (p < 0.05). # indicates statistically significant difference vs 100 N group (p < 0.05). N: NaCl.
(TIF)

S4 Fig Densitometry analyses for Fig 2A, 2B and 2C. Nuclear SIRT6 and total AR expressions were analyzed using densitometry for Fig 2A, 2B and 2C. (A), (B) and (C) corresponds to Fig 2A, 2B and 2C, respectively. Beta-actin (B-actin) was used as total protein loading control, whereas Lamin A/C (Lamin) was used as nuclear loading control. * indicates statistically significant difference vs C group (p < 0.05). # indicates statistically significant difference vs 100 N group (p < 0.05). N: NaCl.
(TIF)

S5 Fig RMSD of protein backbone residues of SIRT1-Ex527 and SIRT6-Ex-527 complexes under high salt (HS) and low salt (LS) conditions. All simulations showed RMSD values converged to a plateau value, indicating suitable simulations for further analysis. Å: Angstrom.
(TIF)

S6 Fig Statistical analyses for MD simulations. (A) Based on RMSF values C atom of Ex-527 molecule, Ex-527 of SIRT6 simulated under high salt condition has significantly reduced fluctuations, thus flexibility, compared with the Ex-5257 molecules of the other simulations. (B) At 20^th^ ns, the distance between center of mass of Ex-527 and center of mass of hydrophobic pocket residues was significantly lower in simulation of SIRT6-Ex-527 complex under high salt, compared with simulations of SIRT1-Ex-527 under high salt and low salt. Moreover, this distance was significantly lower in simulation of SIRT6-Ex-527 complex under low salt, compared with the simulation of SIRT6-Ex-527 complex under high salt, at 20^th^ ns. (C) Based on RMSF values of Cl atom of Ex-527 molecule, Ex-527 atom of SIRT1 under low salt displayed similar fluctuation compared with the Ex-527 of SIRT6 under high salt. * indicates statistically significant difference vs simulation of SIRT6-Ex-527 under high salt (p < 0.05). # indicates statistically significant difference vs simulation of SIRT6-Ex-527 under low salt (p < 0.05). HS: High salt, LS: Low salt.
(TIF)

S7 Fig Legends for the items in Fig 5. (A) Legend for Fig 5A and 5B (B) (C) Legends for Fig 5C. (D) (E) Legends for Fig 5D. C: Carbon, O: Oxygen, N: Nitrogen.
(TIF)
