# Supplementary figures and images for: SIRT6 Is a Positive Regulator of Aldose Reductase Expression in U937 and HeLa cells under Osmotic Stress: In Vitro and In Silico Insights
Source: PLoS One. 2016 Aug 18;11(8):e0161494. doi: 10.1371/journal.pone.0161494 (PMC4990240; doi:10.1371/journal.pone.0161494)

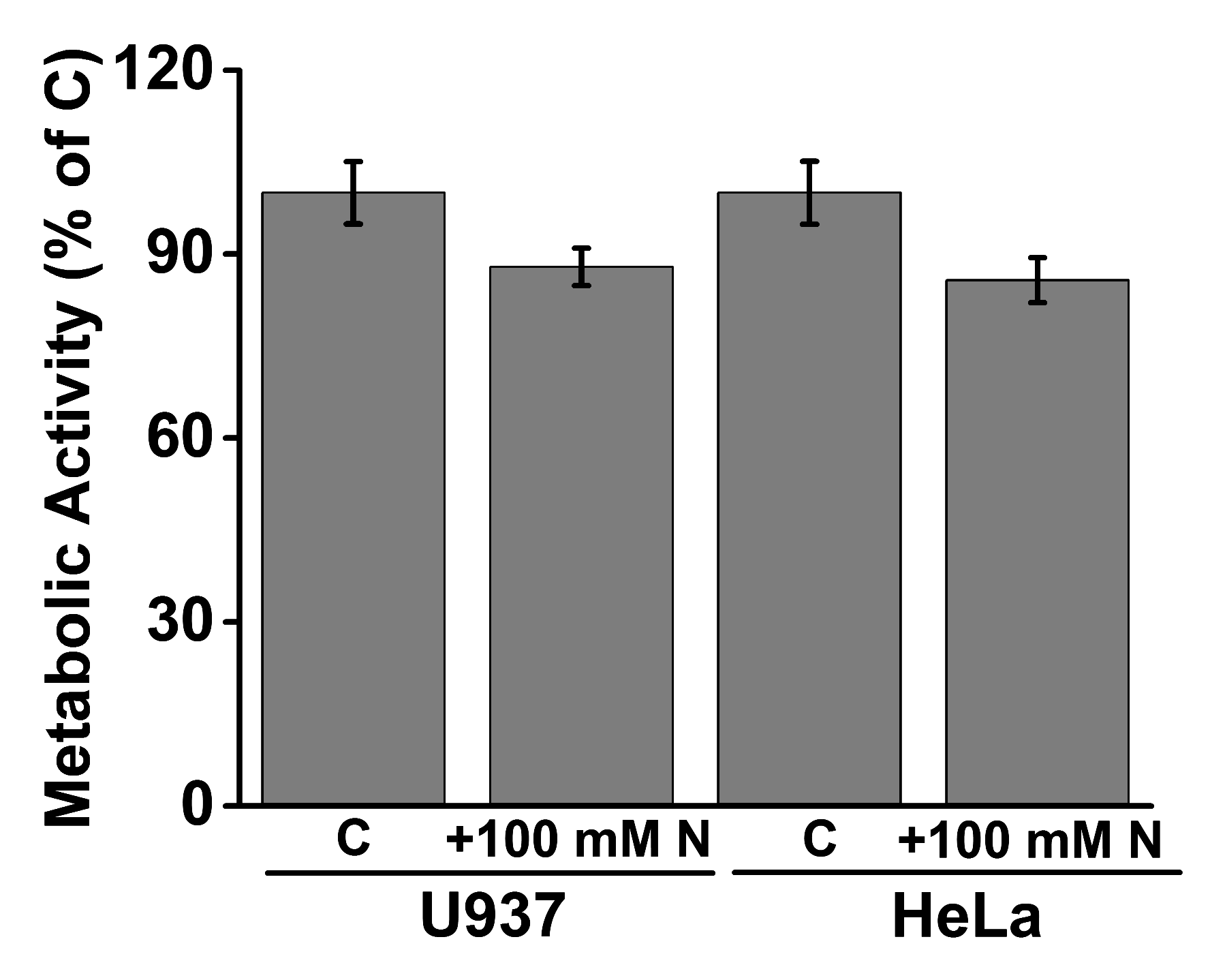

Supplement: S1 Fig — Control (C) indicates U937 cells treated with 5 mM glucose containing SFM for 16 hrs and HeLa cells treated with serum containing medium with 5 mM glucose for 16 hrs. 100 mM N indicates U937 and HeLa cells further treated with 100 mM NaCl (N) for 16 hrs. The reduction in cell viability (~13% for U937 and ~15% for HeLa) was not statistically significant in both cell lines (p > 0.05). Metabolic activity status was analyzed by MTT based colorimetric assay. Average absorbance values of the controls were set to 100%. (TIF) [file pone.0161494.s001.tif]

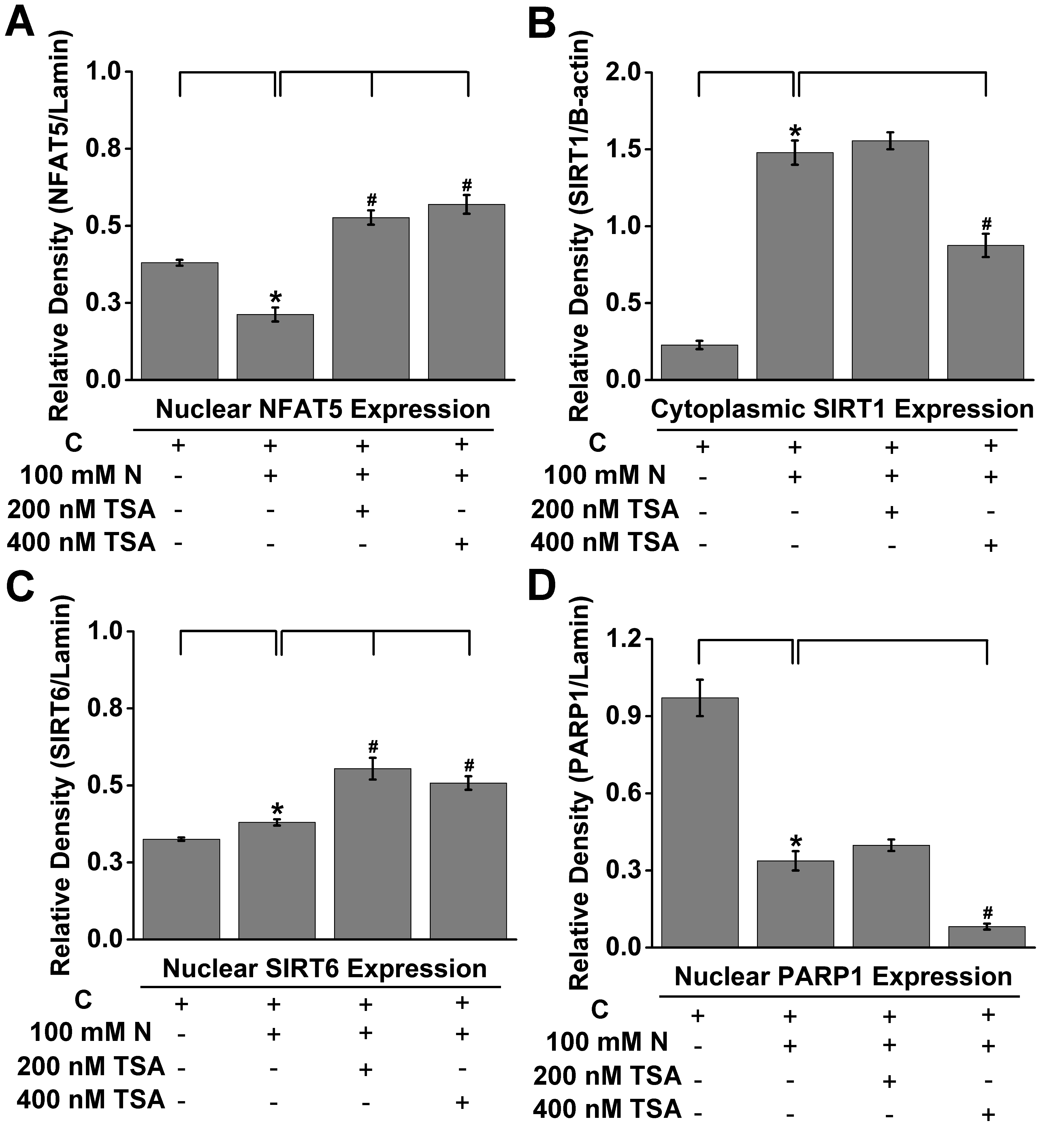

Supplement: S2 Fig — Nuclear NFAT5, SIRT6, PARP1 and cytoplasmic SIRT1 expressions were analyzed using densitometry for Fig 1A. Beta-actin (B-actin) was used as cytoplasmic loading control, whereas Lamin A/C (Lamin) was used as nuclear loading control. * indicates statistically significant difference vs C group (p < 0.05). # indicates statistically significant difference vs 100 N group (p < 0.05). N: NaCl. (TIF) [file pone.0161494.s002.tif]

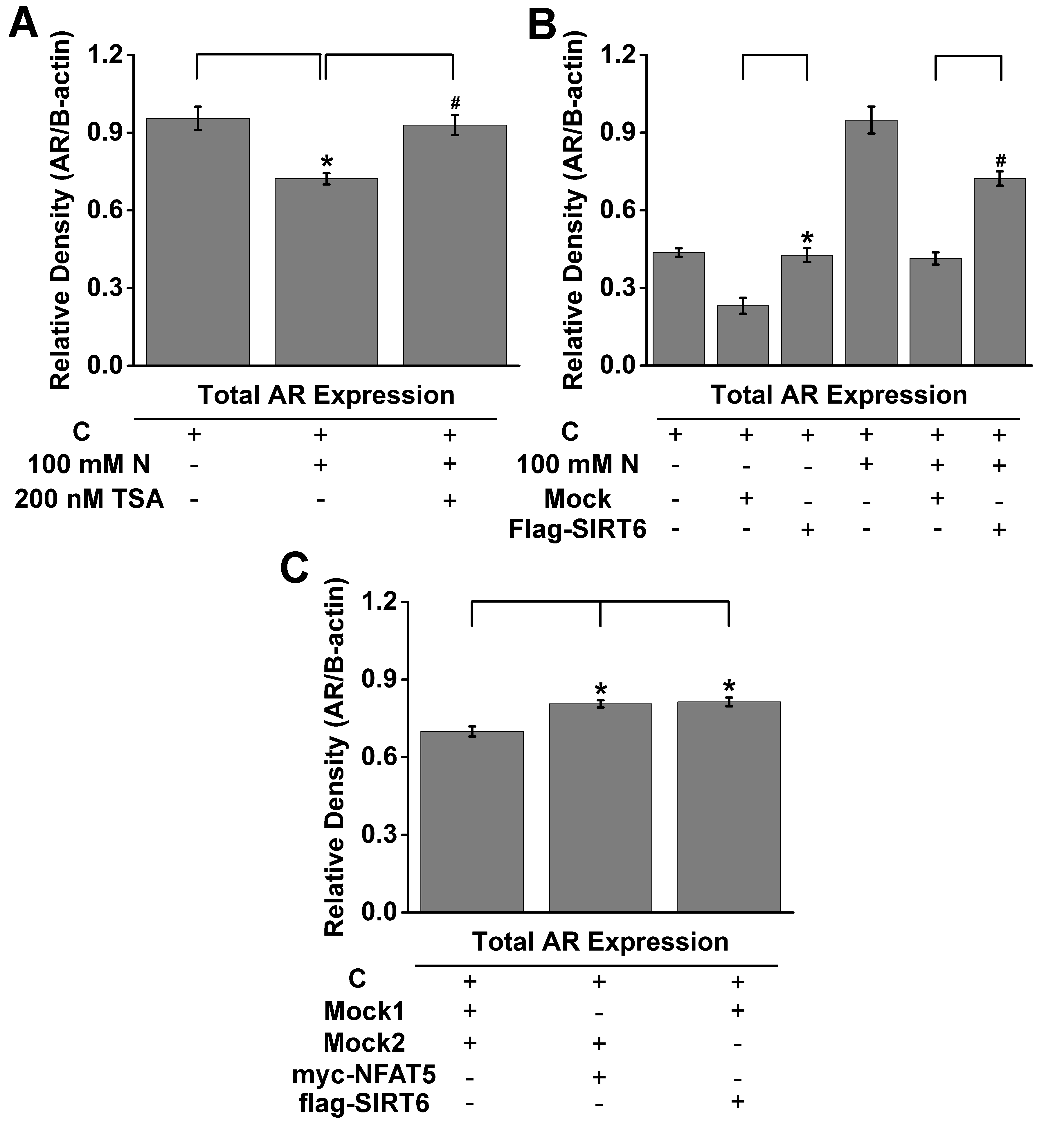

Supplement: S3 Fig — Total AR expressions were analyzed using densitometry for Fig 1B, 1C and 1D. (A), (B) and (C) corresponds to Fig 1B, 1C and 1D, respectively. Beta-actin (B-actin) was used as total protein loading control. * indicates statistically significant difference vs C group (p < 0.05). # indicates statistically significant difference vs 100 N group (p < 0.05). N: NaCl. (TIF) [file pone.0161494.s003.tif]

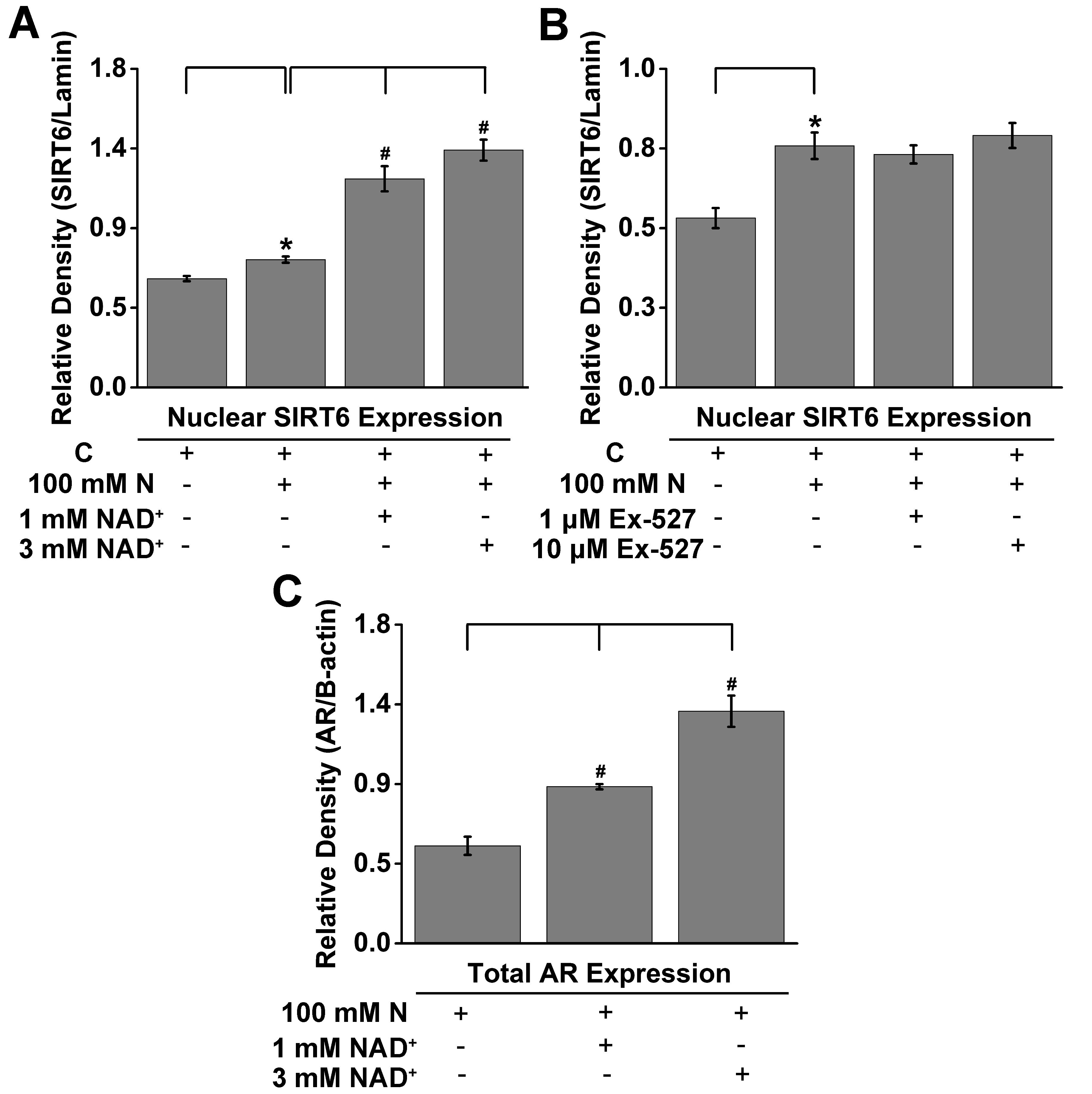

Supplement: S4 Fig — Nuclear SIRT6 and total AR expressions were analyzed using densitometry for Fig 2A, 2B and 2C. (A), (B) and (C) corresponds to Fig 2A, 2B and 2C, respectively. Beta-actin (B-actin) was used as total protein loading control, whereas Lamin A/C (Lamin) was used as nuclear loading control. * indicates statistically significant difference vs C group (p < 0.05). # indicates statistically significant difference vs 100 N group (p < 0.05). N: NaCl. (TIF) [file pone.0161494.s004.tif]

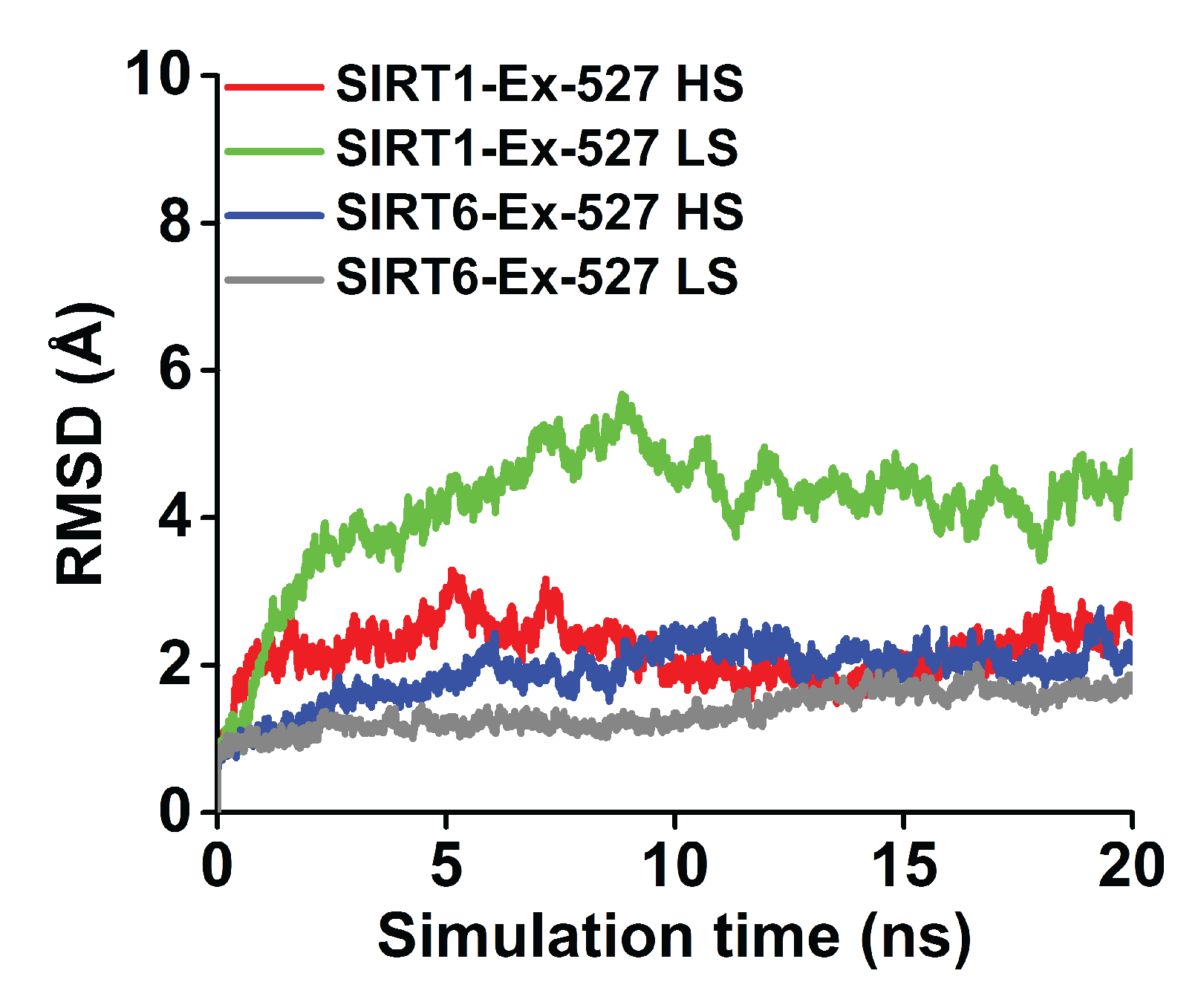

Supplement: S5 Fig — All simulations showed RMSD values converged to a plateau value, indicating suitable simulations for further analysis. Å: Angstrom. (TIF) [file pone.0161494.s005.tif]

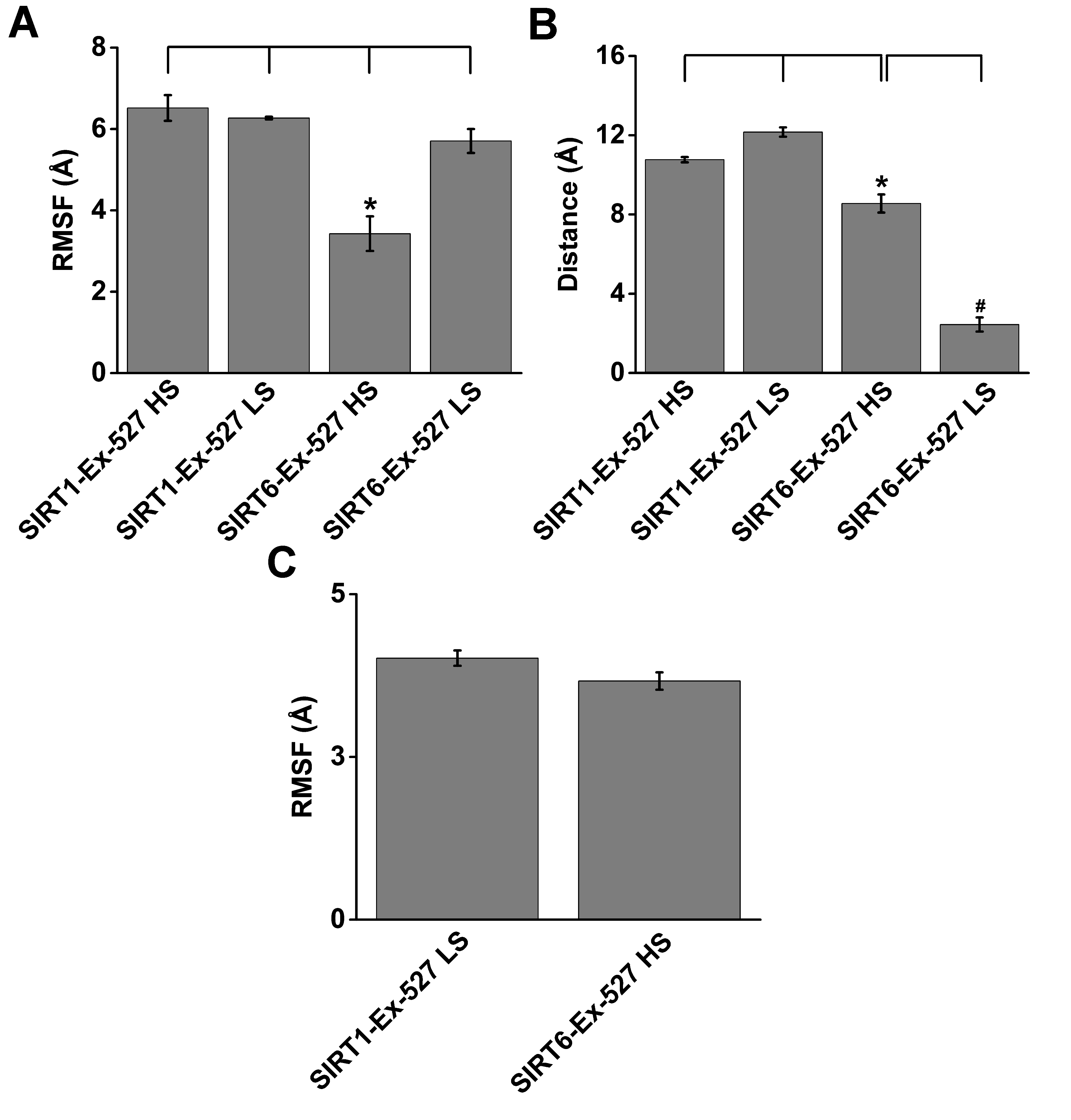

Supplement: S6 Fig — (A) Based on RMSF values C atom of Ex-527 molecule, Ex-527 of SIRT6 simulated under high salt condition has significantly reduced fluctuations, thus flexibility, compared with the Ex-5257 molecules of the other simulations. (B) At 20th ns, the distance between center of mass of Ex-527 and center of mass of hydrophobic pocket residues was significantly lower in simulation of SIRT6-Ex-527 complex under high salt, compared with simulations of SIRT1-Ex-527 under high salt and low salt. Moreover, this distance was significantly lower in simulation of SIRT6-Ex-527 complex under low salt, compared with the simulation of SIRT6-Ex-527 complex under high salt, at 20th ns. (C) Based on RMSF values of Cl atom of Ex-527 molecule, Ex-527 atom of SIRT1 under low salt displayed similar fluctuation compared with the Ex-527 of SIRT6 under high salt. * indicates statistically significant difference vs simulation of SIRT6-Ex-527 under high salt (p < 0.05). # indicates statistically significant difference vs simulation of SIRT6-Ex-527 under low salt (p < 0.05). HS: High salt, LS: Low salt. (TIF) [file pone.0161494.s006.tif]

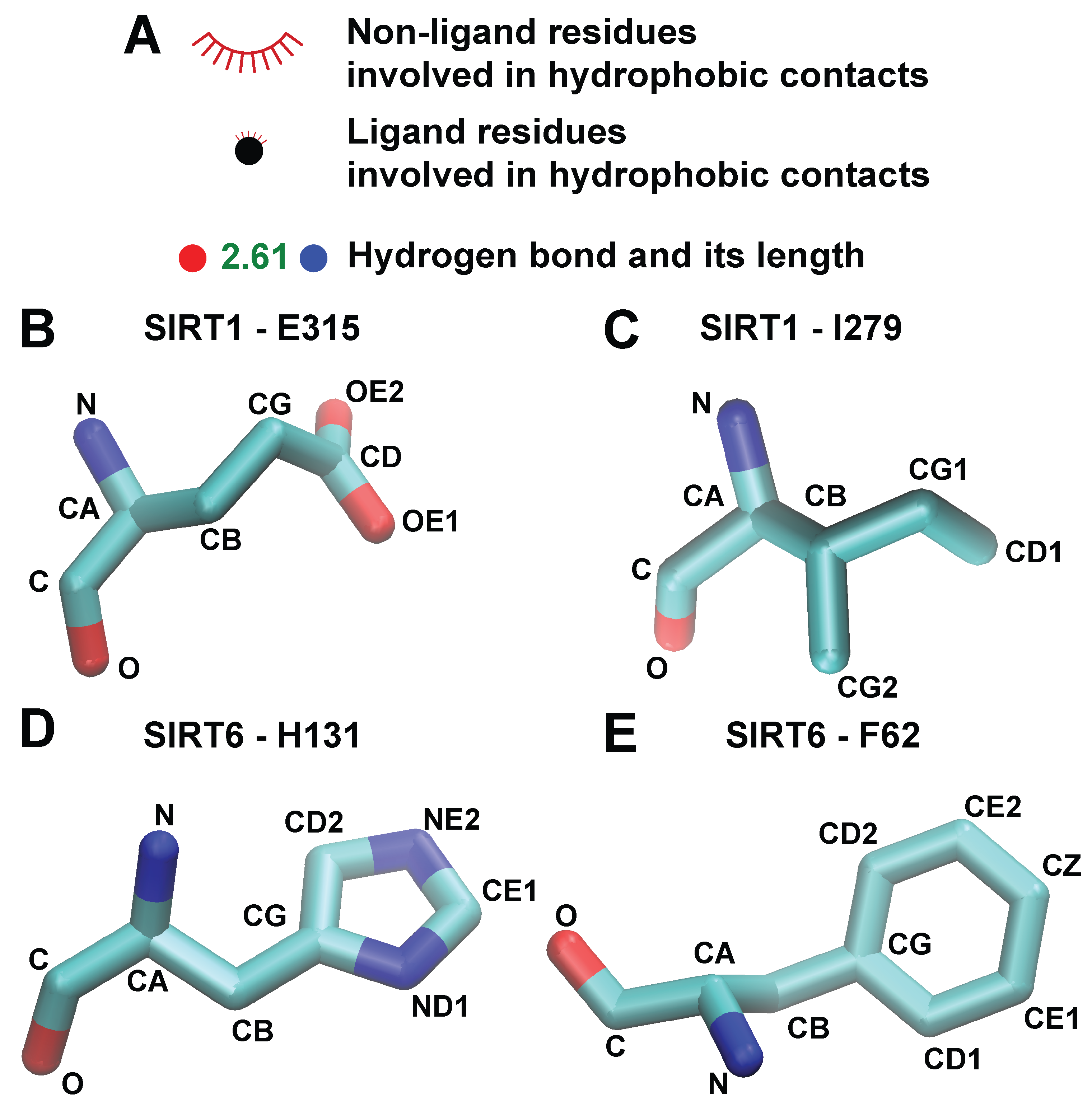

Supplement: S7 Fig — (A) Legend for Fig 5A and 5B (B) (C) Legends for Fig 5C. (D) (E) Legends for Fig 5D. C: Carbon, O: Oxygen, N: Nitrogen. (TIF) [file pone.0161494.s007.tif]
